# A Nanojunction Polymer Photoelectrode for Efficient Charge Transport and Separation

**DOI:** 10.1002/anie.201703372

**Published:** 2017-06-12

**Authors:** Qiushi Ruan, Wenjun Luo, Jijia Xie, Yiou Wang, Xu Liu, Zhiming Bai, Claire J. Carmalt, Junwang Tang

**Affiliations:** ^1^ Solar Energy & Advanced Materials Research Group Department of Chemical Engineering UCL Torrington Place London WC1E 7JE UK; ^2^ Department of Chemistry UCL 20 Gordon Street London WC1H 0AJ UK; ^3^ Key Laboratory of Flexible Electronics (KLOFE) & Institute of Advanced Materials (IAM), Jiangsu National Synergetic Innovation Center for Advanced Materials (SICAM) Nanjing Tech University (NanjingTech) 30 South Puzhu Road Nanjing 211816 P.R. China; ^4^ The school of material science and engineering Beihang University No.37 Xueyuan Road, Haidian district Beijing China

**Keywords:** carbon nitride, nanojunctions, photoanodes, water splitting

## Abstract

A metal‐free photoanode nanojunction architecture, composed of B‐doped carbon nitride nanolayer and bulk carbon nitride, was fabricated by a one‐step approach. This type of nanojunction (s‐BCN) overcomes a few intrinsic drawbacks of carbon nitride film (severe bulk charge recombination and slow charge transfer). The top layer of the nanojunction has a depth of ca. 100 nm and the bottom layer is ca. 900 nm. The nanojunction photoanode results into a 10‐fold higher photocurrent than bulk graphitic carbon nitride (G‐CN) photoanode, with a record photocurrent density of 103.2 μA cm^−2^ at 1.23 V vs. RHE under one sun irradiation and an extremely high incident photon‐to‐current efficiency (IPCE) of ca. 10 % at 400 nm. Electrochemical impedance spectroscopy, Mott–Schottky plots, and intensity‐modulated photocurrent spectroscopy show that such enhancement is mainly due to the mitigated deep trap states, a more than 10 times faster charge transfer rate and nearly three times higher conductivity due to the nanojunction architecture.

Conversion and storage of solar energy into chemical fuels (for example, hydrogen production from water splitting) by a photoelectrochemical (PEC) device has been considered as one of the most promising methods to meet the increasing energy demand of humans.^1^ Substantial efforts have been devoted to developing environmentally friendly, low‐cost, and efficient semiconductor photoelectrodes for such purpose.^2^

Graphitic carbon nitride has been applied to various photocatalytic applications, such as CO_2_ conversion into hydrocarbon fuels,^3^ organic pollutant degradation,^4^ bacterial inactivation,^5^ and water splitting in a suspension system.^6^ It also has great potential to PEC application due to its thermal and chemical stability, appealing band structure, and low cost.^7^ Despite this potential, there are limited reports on its PEC application due to a number of problems, such as a high charge recombination rate, slow charge transfer, and high electrical resistance of the intrinsic graphic carbon nitride.^8^

To overcome these drawbacks, variable strategies have been used during the process of carbon nitride film fabrication, including reducing grain boundary defects of G‐CN via sol processing,^8^ printing carbon nitride on a substrate to build a micro‐contact between carbon nitride and the substrate,^9^ and improving optical and electronic properties of carbon nitride by monomer design.^10^ However, even in the presence of sacrificial agents, the efficiency of the synthesized photoanodes is still very moderate.^10^, ^11^ Elemental doping is an effective strategy to modify the electronic properties of a semiconductor and improve its photocatalytic performance,^12^ which has also been used for carbon nitride.12d, 13 However, a detrimental effect of bulk‐doped semiconductors is the increasing charge recombination centers due to doping,^14^ especially in a PEC system where effective diffusion of charge carriers to the surface is crucial, resulting in a very low efficiency.

As the water splitting reaction takes place on a surface, doping the surface of a photoelectrode can significantly affect the surface state, morphology, surface charge transfer, and electrode/electrolyte interfacial properties^15^ without changing the bulk properties of the photoelectrode. Furthermore, the formation of surface layer/bulk heterojunction may help charge transfer and separation, leading to an improved photocatalytic performance, which has been justified in powder systems.^16^

Herein, for the first time, an efficient one‐step approach has been developed to control the surface layer of metal‐free carbon nitride film fabricated on FTO substrate. Remarkably, the resulting nanojunction film, composed of 100 nm B‐doped carbon nitride and 900 nm bulk carbon nitride, exhibits 10 times higher PEC performance than G‐CN film. We further investigated the effect of the boron‐doped carbon nitride nanolayer on interfacial charge transport and recombination, which supports the extraordinary photocatalytic activity of the nanojunction photoanode.

A new rapid vapor deposition method was developed to prepare the G‐CN film on FTO glass, resulting into the optimized thickness of ca. 1000 nm (Supporting Information, Figure S1). In a typical run, a precursor disk filled with dicyandiamide (DCDA) was placed above FTO glass, which was then put into a 600 °C pre‐heated furnace for 20 minutes and cooled down to room temperature in air. The boron‐doped top nanolayer and G‐CN bulk layer was prepared by modifying the vapor deposition method as shown in the Supporting Information, Scheme S1, where DCDA was replaced by a mixture of boric acid and DCDA (more details in the Supporting Information). The XRD pattern of both s‐BCN and G‐CN were very similar, indicating that the bulk of the film was G‐CN and that the top layer of s‐BCN is too thin to be observed by XRD (Supporting Information, Figure S2 a). The chemical environment of boron in the top layer of s‐BCN was determined by X‐ray photoelectron spectroscopy (XPS). In Figure 1 a, a single B 1s peak centered at 191.7 eV with a FWHM of 1.56 eV was detected, which can be assigned to a B−N−C bond connection.^17^ The binding energy of boron in s‐BCN is higher than that of h‐BN (190.1 eV),^18^ suggesting boron atoms in s‐BCN structure are more electropositive. This can be explained by one boron atom bonding with three nitrogen atoms in the heptazine.17a XPS B 1s peak of B−C bonds was usually centered at 189.4 eV,17a which is absent in the s‐BCN structure. The boron content in s‐BCN surface layer could be controlled by adjusting the boron amount in the precursor. The optimum s‐BCN sample had a real boron content of 0.6 % on the surface layer, as determined by XPS. To investigate the structural details of boron incorporation into CN matrix, ^11^B chemical shift data was obtained with BF_3_ Et_2_O as the reference. ^11^B Solid‐state MAS NMR spectra shows one single peak at −5.93 ppm which can be assigned to boron substituting “bay‐carbon” site^19^ (Figure 1 b; Supporting Information, Figure S3). The negative chemical shift indicates the stronger shielding effects and increased electron cloud density around the B nuclei, which can be explained by electrons flowing to B atom by forming conjugated π bonds with surrounding three N atoms. XPS and NMR spectra indicate that boron atoms have most likely substituted for carbon in CN_2_(NH_2_) cluster. STEM elemental mapping in the Supporting Information, Figure S4 demonstrates the evenly distributed boron doping on the surface. XPS depth profile of B 1s (Figure 1 a) shows that the boron concentration drops with depth. Negligible boron signal can be observed at 100 nm below the surface, suggesting that boron doping is limited to the 100 nm surface of s‐BCN. The fabrication process of the s‐BCN film was further detailed in the Supporting Information by observation of samples synthesized for 5 min, 10 min, 15 min, and 20 min (Supporting Information, Figure S5), which evidences the evolution from bulk G‐CN to the s‐BCN photoanode.

**Figure 1:**
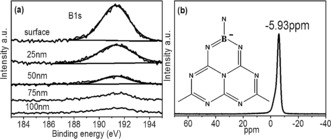
a) XPS depth profile of B 1s in s‐BCN film. b) Solid‐state ^11^B MAS NMR spectra of s‐BCN.

The optical properties of G‐CN and s‐BCN film were investigated by UV/Vis spectroscopy, shown in the Supporting Information, Figure S6a with UV/Vis spectra of bulk G‐CN and bulk B‐doped CN powder in Figure S6 b as a reference. Pure carbon nitride exhibits a band gap of ca. 2.72 eV, similar to previous reports.^20^ S‐BCN shows a red shift of the spectrum with a slightly narrower band gap of ca. 2.65 eV. The narrowed band gap of s‐BCN extends its visible light absorption, which is somehow beneficial to photocatalytic reaction. The N 1s XPS spectrum shows an additional peak at 399.6 eV, which is believed to be associated with boron substitution (Supporting Information, Figure S2 c). The unchanged carbon chemical environment (Supporting Information, Figure S2 d) further proves boron atoms substitute carbon not nitrogen. Both FTIR and Raman spectrum also confirm that s‐BCN has a typical local structure of G‐CN (Supporting Information, Figure S7).

The photocatalytic water splitting by these samples in a PEC cell was then carried out and the anodic photocurrent curves of G‐CN and s‐BCN are displayed in Figure 2 a. G‐CN and s‐BCN samples show negligible dark current from 0.4 V to 1.23 V vs. RHE. G‐CN shows a low photocurrent of 10.6 μA cm^−2^ at 1.23 V vs. RHE, similar to previous reports.^8^, ^9^, ^11^ The performance of the carbon nitride samples was significantly enhanced by adding a nanolayer of boron doped CN. To find the optimum boron content, different samples were tested (if *a* wt % boric acid precursor and (1−*a*) wt % DCDA precursor were used, the prepared sample was denoted s‐BCN(a%); Figure 2 a). The s‐BCN(4 %) nanojunction film exhibits the highest photocurrent of 103.2 μA cm^−2^ at 1.23 V vs. RHE, which is the highest for g‐CN based polymer photoanode in the absence of any chemical scavenger, to the best of our knowledge (a summary of PEC performance of CN based polymer photoanodes is given in the Supporting Information, Table S1). This specific film has a 100 nm surface doped CN layer onto 900 nm G‐CN. The real boron concentration on the doped CN layer was determined to be 0.6 % by XPS. Further increases of boron concentration resulted in a decrease in photocurrent. To investigate the photocatalytic response of electrodes at different wavelengths, incident light‐to‐electron conversion efficiency (IPCE) measurements of the both s‐BCN(4 %) and G‐CN films were acquired at 1.23 V_RHE_ (Figure 2 b). The IPCE of G‐CN was measured to be around 1 % at 400 nm, while remarkably increasing to nearly 10 % for s‐BCN(4 %) at the same wavelength.

**Figure 2:**
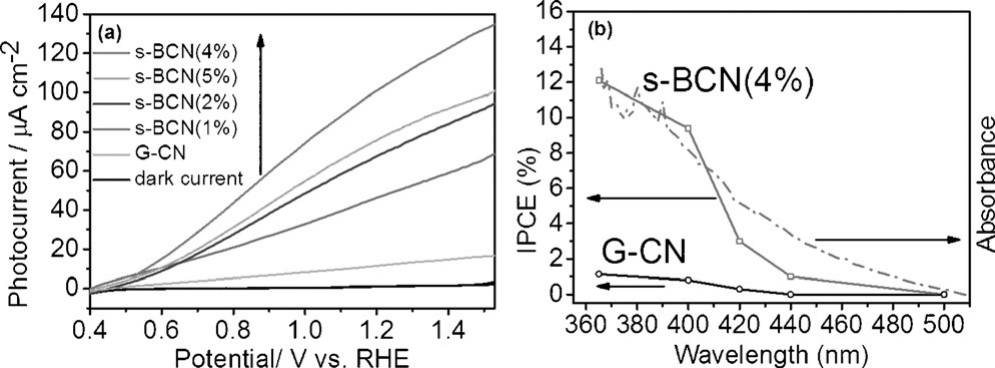
a) Photocurrent–potential curves for s‐BCN (0–5 %) illuminated from the back side; electrolyte: 0.1 m Na_2_SO_4_ solutions (pH 6.5), one‐sun irradiation provided. b) IPCE plot of G‐CN and s‐BCN (4 %) at 1.23 V vs. RHE.

More electrochemical analysis of s‐BCN and G‐CN samples were carried out, including Mott–Schottky (MS) plots and electrochemical impedance spectroscopy (EIS) Nyquist plots, to investigate the reason of dramatically enhanced IPCE. Typical MS plots in the dark condition disclose the n‐type characteristics of s‐BCN and G‐CN owing to the positive slope of the linear plots (Figure 3 a).^21^ The carrier density of s‐BCN and G‐CN derived from Mott–Schottky equation are of the same order of magnitude. The similar carrier density suggests that nanoscale surface doping, as expected, does not affect the carrier concentration in the bulk. The flat‐band potential was also derived from the extrapolation of MS plots at different frequencies (Supporting Information, Figure S8). For both G‐CN and s‐BCN, the flat‐band potentials are determined to be −1.30 V vs. Ag/AgCl (pH 6.5), consistent with previous reported pristine carbon nitride.^21^, ^22^ Given the band gap obtained from UV/Vis spectra, the valence bands of G‐CN and BCN were determined to be +1.42 V and +1.35 V vs. Ag/AgCl, respectively. Apparently, boron doping shifts the valence band of carbon nitride upward. The driving force between valence bands of carbon nitride and boron doped nanolayer enables the holes transfer from the bulk to the surface for photooxidation reaction. EIS Nyquist plots (Figure 3 b) were used to observe the charge transfer rate. A significantly decreased diameter for semicircle of s‐BCN was observed. The electron‐transfer conductivity calculated with an equivalent circuit shows a three time increase for the s‐BCN sample compared with G‐CN, which is further confirmed by the measurement of the photocurrent (inset of Figure 3 bb). A remarkable improvement in photocurrent of s‐BCN over G‐CN is likely due to a more efficient charge transfer for s‐BCN samples.

**Figure 3:**
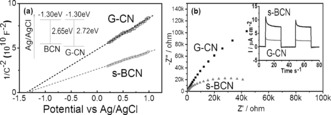
a) Mott–Schottky plots of G‐CN and s‐BCN at 1 kHz frequency (plots at other frequencies shown in the Supporting Information, Figure S8). b) Nyquist plots of G‐CN and s‐BCN obtained by applying a sine wave with amplitude of 5.0 mV over the frequency range from 10 kHz to 0.1 Hz, with the inset showing the periodic on/off photocurrent response of G‐CN and s‐BCN electrodes in 0.1 m Na_2_SO_4_ with 0 V bias versus Ag/AgCl.

To further investigate kinetics of charges in s‐BCN, intensity modulated photocurrent spectroscopy (IMPS) was employed. Typical IMPS responses of G‐CN and s‐BCN photoanode were provided in a complex plane (Supporting Information, Figure S9). The high frequency semicircle (lower semicircle) indicates charge transport and relaxation in photoanode, whose intercept with *x*‐axis equals to the hole current without recombination, while the low frequency semicircle (upper semicircle) corresponds to the competition between interfacial charge transfer and surface recombination.^23^ Compared with G‐CN, the larger hole current (no recombination) of s‐BCN implies more efficient hole capture by surface, which in sequence are either transferred to electrolyte or recombine with electrons.^24^ Kinetics of charges can be represented by the first order rate constant of surface recombination (*K*_r_) and interface charge transfer (*K*_t_) derived from IMPS plot data (Supporting Information, Figure S10) and plotted in Figure 4 a. Larger *K*_r_ describes a faster charge recombination on the electrode surface. In both samples, *K*_r_ decreases with potential as expected for an ideal semiconductor/electrolyte interface, because large potential increases the band bending so that charge recombination both in the bulk and at surface is suppressed.^24^ It is noted that at low potential, *K*_r_ in s‐BCN was larger than that in G‐CN. It can be explained that boron doping can introduce defects on the surface that becomes recombination centers for charge recombination. Consistent with this, a too high concentration of boron doping promoted surface recombination and resulted in poorer PEC performance (Figure 2 a), so the bulk doped samples always yielded a low photocurrent as reported.^25^ However at high potential, the surface recombination was suppressed as most electrons were driven to the counter electrode by bias. The sharper decay of K_r_ in s‐BCN implies the mitigated deep trap states compared with that in G‐CN. *K*_t_ is used to describe the transfer of charges between electrode and electrolyte, that is, reaction between holes and water in the study. As shown in Figure 4 a, *K*_t_ of s‐BCN samples is apparently 10 times larger than that of G‐CN (for example, 7 s^−1^ for s‐BCN samples and 0.7 s^−1^ for G‐CN at 0.2 eV vs. Ag/AgCl), indicating nearly 10 times faster reaction rate of photoholes with water as a result of the nanojunction architecture. More importantly, the ratios of *K*_t_/*K*_r_ of G‐CN and s‐BCN which are plot in Figure 4 a (inset) indicate the efficiency of the photoanode. At potential larger than 0.2 eV vs. Ag/AgCl, the value of *K*_t_/*K*_r_ of s‐BCN becomes sharply increase, much larger than that of G‐CN, that is, three times larger at 0.4 eV vs. Ag/AgCl, indicating much more charges to be used for water oxidation by the nanojunction photoanode.

**Figure 4:**
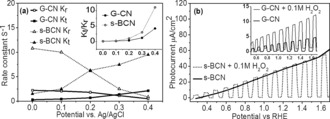
a) Potential dependence of the rate constant *K*_t_ and *K*_r_ for s‐BCN and G‐CN samples. Illumination: 365 nm UV light; b) photocurrent of G‐CN and s‐BCN (inset) with and without addition of 0.1 m H_2_O_2_, pH 5.7.

To further confirm the contribution of efficient charge separation due to the nanojunction, hole separation from electrons was observed by adding the widely used hole scavenger hydrogen peroxide (H_2_O_2_).^26^ As shown in Figure 4 b, adding H_2_O_2_ significantly improves photocurrent of G‐CN film, indicating that charge separation on the G‐CN surface is less efficient, consistent with the reported.20a, 27 Increase of the photocurrent of s‐BCN film is negligible after adding H_2_O_2_ as hole scavenger, which indicates that an efficient charge separation was achieved by the nanojunction of the s‐BCN photoanode.

Based on the calculated band alignment in the inset of Figure 3 a, boron doping shifts the valence band of CN upward. The driven force between valence band of G‐CN layer and upshifted valence band of B‐doped CN layer allows holes after excitation to transfer from G‐CN to B‐doped CN layer, while electrons transfer to the G‐CN layer even in the presence of very weak bias. It is further proved by the experiments of H_2_O_2_ influence that efficient charge separation occurring at G‐CN/BCN interface allows more holes transport to the surface with dramatically mediated recombination. A 10 times higher charge transfer rate on the s‐BCN top layer further helps the fast water oxidation reaction. Therefore the final photon conversion efficiency in the visible region (at 400 nm) enhances by a factor of 10 owing to the nanojunction.

To conclude, we have presented a novel strategy for the production of nanojunctions on carbon nitride film, which acts as an efficient photoanode for water oxidation. The resulting nanojunction film shows a record photocurrent density of 102.3 μA cm^−2^ at 1.23 V vs. RHE under one sun condition and an extremely high IPCE of 10 % at 400 nm, which is 10 times higher than that of pristine carbon nitride. Data from XPS, UV/Vis, and PEC show that the surface nanolayer doping can significantly improve the solar to fuel conversion efficiency but without dramatically changing the band gap and bulk properties. Such enhancement is mainly due to the efficient charge separation, fast charge transfer and mitigated deep trap states in the nanojunction, as evidenced by EIS, MS, and IMPS spectroscopy. Furthermore, the H_2_O_2_ addition confirms that the efficient charge separation can be achieved by the nanojunction itself. We believe that this surface nanojunction strategy can be extended to other semiconductors to efficiently improve their applications in the fields of solar fuel conversion and environmental purification.

## Conflict of interest

The authors declare no conflict of interest.

## Supporting information

As a service to our authors and readers, this journal provides supporting information supplied by the authors. Such materials are peer reviewed and may be re‐organized for online delivery, but are not copy‐edited or typeset. Technical support issues arising from supporting information (other than missing files) should be addressed to the authors.

Supplementary
