# Supplementary material for: A Nanojunction Polymer Photoelectrode for Efficient Charge Transport and Separation
Source: Angew Chem Int Ed Engl. 2017 Jun 12;56(28):8221–5. doi: 10.1002/anie.201703372 (PMC5519949; doi:10.1002/anie.201703372)
Supplement: Supplementary file 1 — Supplementary [file ANIE-56-8221-s001.pdf]

Angewandte Chemie
Eine Zeitschrift der Gesellschaft Deutscher Chemiker

Supporting Information

A Nanojunction Polymer Photoelectrode for Efficient Charge Transport and Separation

*Qiushi Ruan, Wenjun Luo, Jijia Xie, Yiou Wang, Xu Liu, Zhiming Bai, Claire J. Carmalt, and Junwang Tang**

anie_201703372_sm_miscellaneous_information.pdf

Supporting Information
y

WILEY-VCH

Experimental Procedures

Synthesis methods

G-CN and s-BCN films were synthesized by a new “rapid thermal vapour deposition” method. 50mg Dicyandiamide (Alfa Aesar, 99%) and 0wt%-5wt% Boric Acid (Sigma-Aldrich, 99.5%) were mixed and dissolved with DI water in a Φ35mm petri dish. After drying at 70°C for 1h, the mixture of Dicyandiamide and Boric Acid were crystallized and adhered to the internal surface of the petri dish. A piece of 20mm×20mm FTO glass used as the substrate was placed on a slightly concave crucible lid and covered by the petri dish. The sample was calcined in a 600°C preheated Muffle furnace (Carbolite, CWF 1300) for 20min and quenched to the room temperature in air.

Characterisation

XRD patterns were taken by a D8 Bruker Diffractometer. UV-Vis absorption spectra were collected using a Shimadzu UV-Vis 2550 spectrophotometer fitted with an integrating sphere using $BaSO_4$ as the reference material. FTIR spectroscopy was performed on a Perkin-Elmer 1605 FT-IR spectrometer in the wavenumber range from 400 – 4000 cm^{-1} with a resolution of 0.5 cm^{-1}. Raman spectroscopic measurements were performed on a Renishaw InVia Raman Microscope, using a 325nm excitation laser, and a wavenumber range from 100 – 3000 cm^{-1}. XPS measurements were done on a Thermoscientific XPS K-alpha surface analysis machine using an Al source. Analysis was performed on the Casa xps software. SEM images were taken by a JOEL JSM-7401F Scanning Electron Mictoscope. TEM measurements were taken using a JEOL2010F. 11B NMR was acquired with a solid state NMR spectrometer (Bruker), using BF_3Et_2O as the reference.

Photocatalytic analysis

The photoelectrochemical properties were investigated in a conventional three-electrode cell using an electrochemical analyzer (IVIUM Technologies). The as-prepared film, a Pt net and an Ag/AgCl electrode were used as the working, counter and reference electrodes, respectively. Sunlight was simulated with a 150 W xenon lamp (Newport) and AM 1.5 filter (Newport). The light intensity was set using a calibrated crystalline silicon solar cell, equivalent to global AM 1.5 illumination at 100 mW/cm^2. The photocurrent of samples was measured in 0.1M Na_2SO_4 aqueous solution (pH=6.5). Samples were illuminated from the back side (FTO substrate/semiconductor interface) and the mask-off irradiated area was 0.28 cm^2. Cyclic voltammetry measurements were performed at a scan speed of 10mV s^{-1}. The potentials of the working electrodes can be calculated by the formula $V_{RHE}=V_{Ag/AgCl}$ + 0.059pH + 0.1976V, where VRHE is a potential vs. a reversible hydrogen potential, $V_{Ag/AgCl}$ is a potential vs. Ag/AgCl electrode. The Mott–Schottky curves were measured at a certain DC potential range with an AC amplitude of 5 mV and a frequency of 1000Hz under dark condition. Electrochemical impedance spectra(EIS) were measured at 0.0 VAgCl/Ag. A sinusoidal ac perturbation of 5mV was applied to the electrode over the frequency range 0.1Hz−10kHz. Intensity modulated photocurrent spectroscopy (IMPS) measurements were conducted using a potentiostat (IVIUM technology) in a three-electrode configuration. Modulated illumination (LED: ultraviolet 365nm) was provided by a ModuLightmodule (IVIUM technology). The IPCE was obtained under light irradiation using a different wavelength generated by monochromatic filters with a 10 nm band width. The following equation was used for IPCE calculation[1]:

$$\boldsymbol{IPCE = \frac{1240 * I_{ph}}{P * \lambda}}$$

Where P and λ are incident light intensity (μW cm^{-2}) and wavelength (nm), respectively; I_{ph} is the photocurrent density (μA cm^{-2}).

Results and Discussion

WILEY-VCH

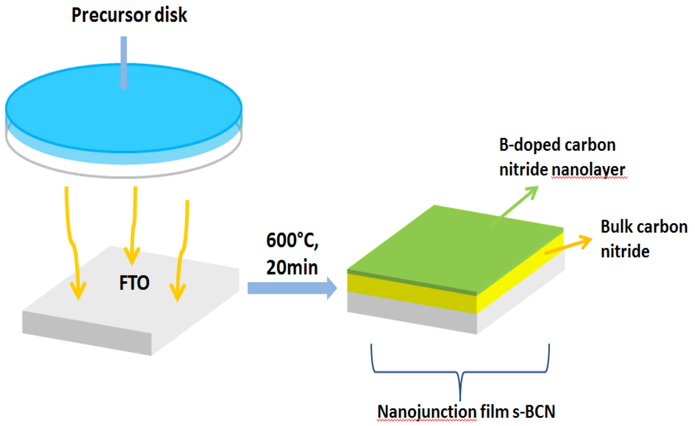

Scheme S1. Illustration of one step depositing s-BCN on FTO substrate.

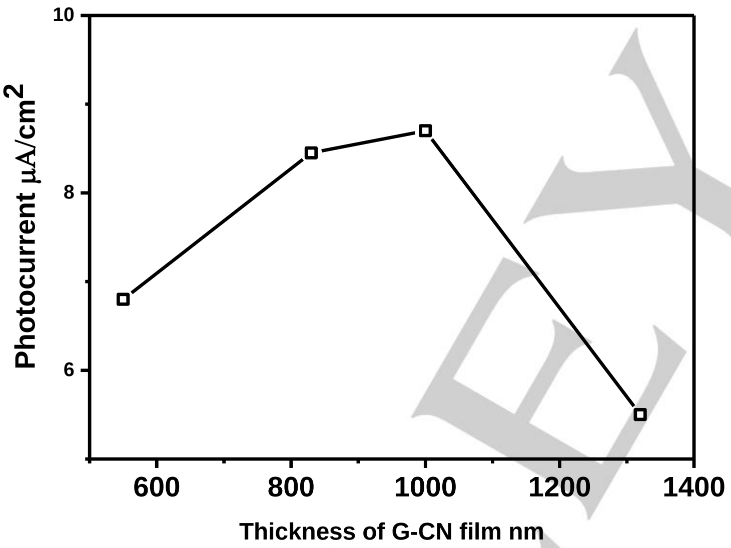

Figure S1. PEC performance of G-CN film at 1.23V_{RHE} with its thickness.

We optimized the thickness of G-CN film to achieve the best PEC performance before we started doping boron on the surface. The relationship between G-CN film thickness and its PEC performance (photocurrent at 1.23V_{RHE}) was shown in Figure S1. The optimum thickness of G-CN film by this method is around 1μm. Thicker film leads to a lower PEC performance which is probably due to a longer charge transfer distance in the film. Thinner film also causes detrimental effect on the photocatalytic performance as light absorption can become less efficient.

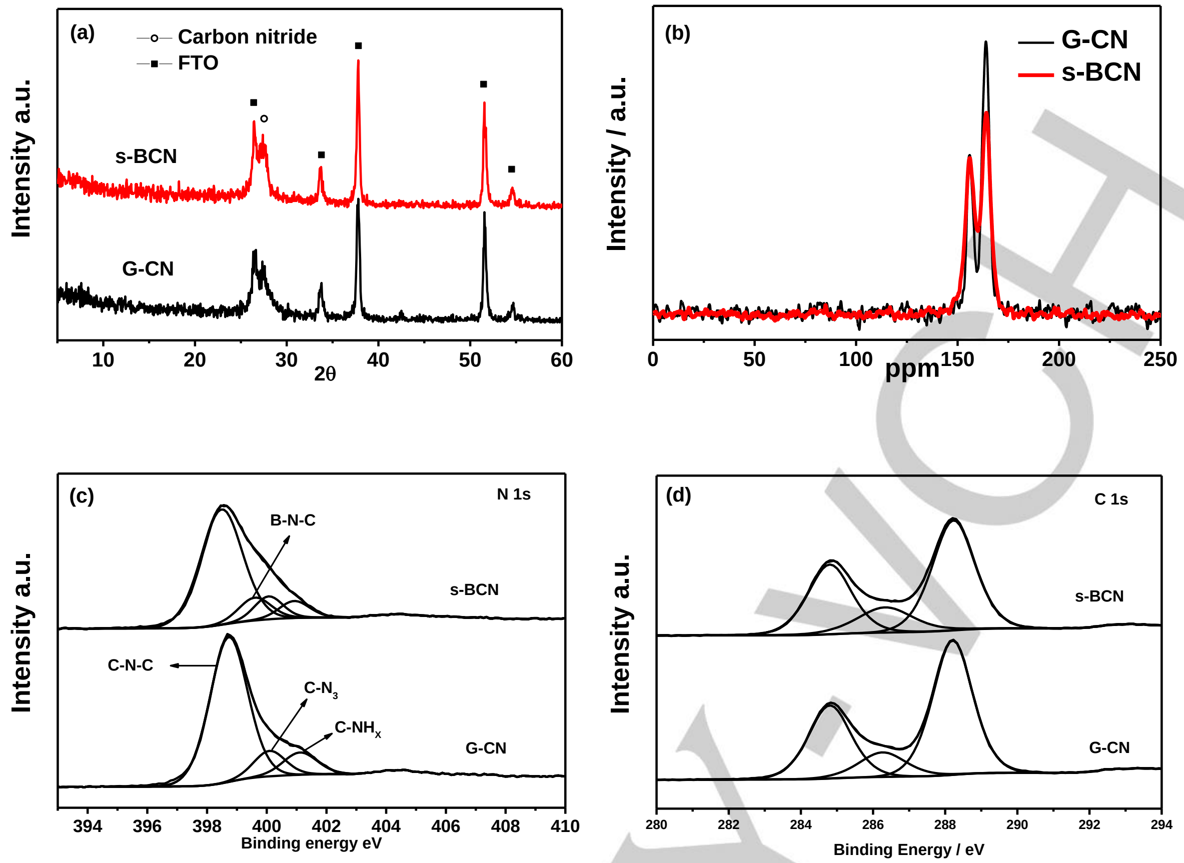

Figure S2 a) XRD pattern and b) Solid state ^{13}C CP-MAS spectra of G-CN and s-BCN; c) N1s XPS spectra and d) C1s XPS spectra of G-CN and s-BCN

Solid-state ^{13}C CP-MAS NMR spectra (Figure S2b) shows two distinct peaks at δ = 156.0 ppm and δ = 164.1ppm, respectively. The first peak is attributed to the sp^3 hybridized C centered in the melem unit. The second peak can be assigned to the surround C atoms in $CN_2(NH_2)$ group[2]. N1s XPS spectrum of s-BCN sample was fitted to four components in Figure S2c. The main peak located at 398.7 eV was attributed to C-N-C bonds[3]. The other two peaks at higher energy account for C-$[N]_3$ (400 eV) and C-NH_x bonds (401.1 eV) respectively, consistent with pure carbon nitride.[3] There is an additional peak at 399.6 eV, which is believed to be associated with boron substitution. Replacing carbon with boron would introduce a positive charge in the structure, which will shift the binding energy of nitrogen in B-N-C bonds to a higher level. Further evidence can be found in Figure S5e and S5f. During the process of calcination, N1s XPS spectra show a shoulder around 399.6 eV, which increases along with boron concentration. Figure S2d shows C1s XPS peaks at 288.2eV, corresponding to the binding energy of C-N-C bonds. The small peak at 286.4 eV corresponds to C-O bonds and the peak centered at 284.8eV is a C-C carbon impurity. C1s XPS spectra of G-CN and s-BCN exhibit no difference. The unchanged carbon chemical environment further proves that boron atoms substitute carbon not nitrogen.

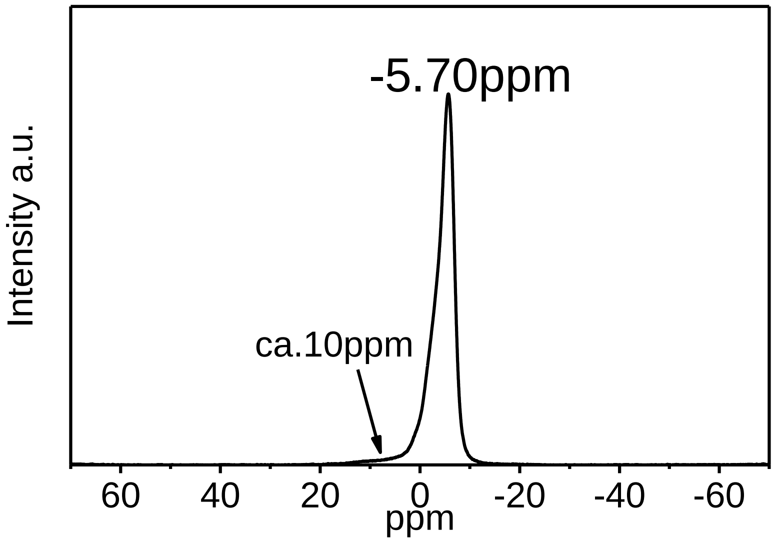

Figure S3 11B MAS NMR spectra of $CNB_{0.04}$, following Wang et al's receip[4].

It is noticed that in the previous report[4], the incorporation of boron into bay-carbon site was assigned to the peak at -4.99ppm in ^{11}B solid-state MAS NMR spectra. The 1ppm difference could be due to different reference materials they used, which was not mentioned in the report. To verify it, we followed their recipe to produce $CNB_{0.04}$ products and ran ^{11}B solid state MAS NMR on the sample using BF_3 Et_2O as the reference (To keep consistent with our sample, only a small amount of 4wt% B source was added). In Figure S2, one strong peak at -5.70ppm was observed, very similar to ours (-5.93ppm), which was ascribed to boron incorporating into "bay-carbon" in the paper[4]. This verifies our results.

It is also noticed that in the same paper[4], a small peak at 10.13ppm for 11B NMR spectrum was observed, which was suggested as boron incorporating into corner carbon sites. However, in our s-BCN sample, we observed only one boron position (boron into bay carbon sites). This difference in boron doping positions might cause different electronic features and flat band position between the CNB and our s-BCN.

WILEY-VCH

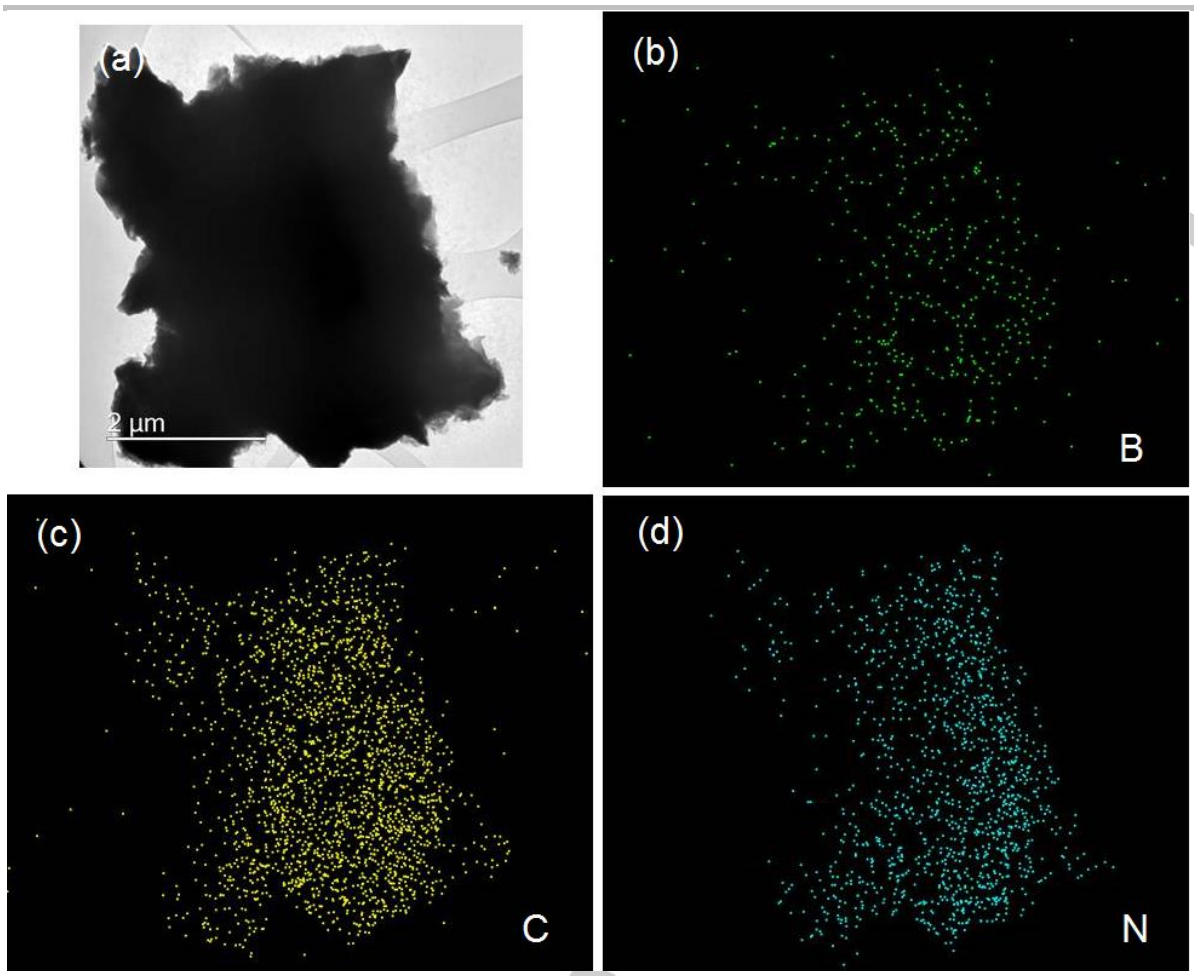

Figure S4 a) TEM top view of s-BCN sample (the sample flake was scraped from the FTO substrate), b-d) boron, carbon and nitrogen elemental mapping.

With a STEM, the distribution of B along with C and N from the top view of s-BCN sample flake was observed and shown in Figure S4. The distribution of boron fits well with carbon and nitride, indicating the evenly doping of boron on the surface.

WILEY

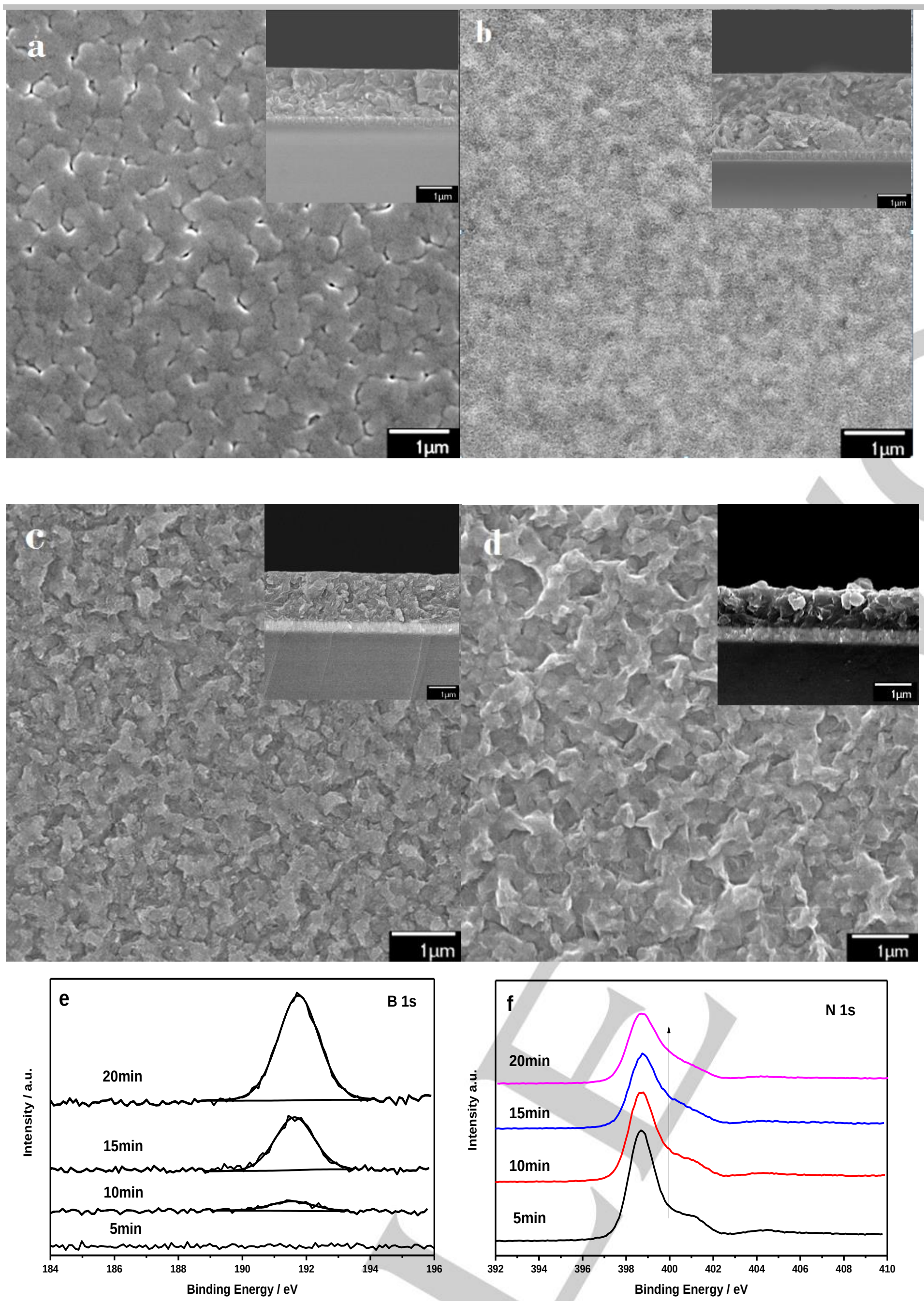

Figure S5 SEM top and cross section (inset) view of s-BCN calcined for a) 5min, b) 10min, c) 15min, d) 20min and their e) B1s and f) N1s XPS spectrum

To further investigate the fabrication process of boron doping into the carbon nitride film, s-BCN samples synthesized for 5 min, 10 min, 15 min and 20 min were characterized by SEM and XPS (Figure S5). Clear differences were observed among samples synthesized at different times in terms of morphology, thickness and boron concentration. A transformation of morphology with calcination time was observed, from a loosely compact surface to a rough and dense morphology. A synthesis time of 5 minutes produced a 1.5 μm-thick carbon nitride film on the substrate with negligible boron appearing on the surface. This is due to the process of boron doping being much slower than carbon nitride polymerization. At 10 mins, the carbon nitride film grew to 2.2 μm and small boron peaks appeared in the B1s XPS spectrum. This suggests that further polymerization of carbon nitride with boron doping was taking place at the same time. At 15 mins, the thickness of the carbon nitride film was reduced to 1.7 μm and further to 1.0 μm (100 nm B doped carbon nitride and 900 nm carbon nitride) at 20 mins, while boron doping concentration on the surface continued to increase. The reduction in thickness was most likely due to polymerization of carbon nitride and decompostion of a less stable structrue. The accumulation of boron on the surface implies that the good thermal stability of boron doped carbon nitride is probably due to nitrogen being stabilized by bonding to the boron atoms[5].

WILEY-VCH

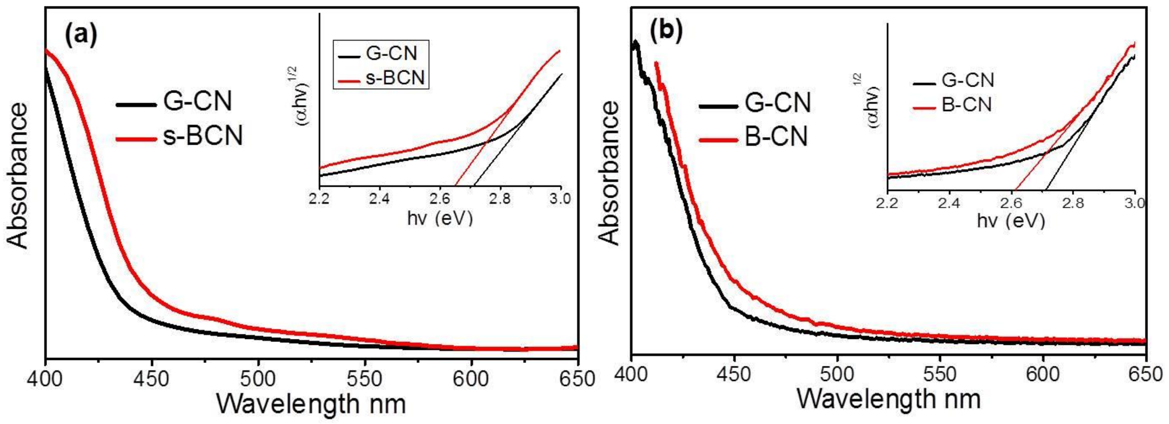

Figure S6 UV-Vis spectrum and tauc plot (inset) of a) G-CN and s-BCN film; b) bulk G-CN and bulkB-CN powder.

UV-Vis spectra on g-CN film, s-BCN film, bulk G-CN powder and bulk B-doped CN powder are shown in Figure S6. It is found that bulk B-doped CN powder has a band gap of 2.61eV, smaller than that of bulk G-CN powder (2.72eV). It is noticed that the band gap measured for s-BCN film (2.65eV) (composed of 100nm B-doped CN layer and 900nm G-CN layer) is larger than that of bulk B-doped CN (2.61eV) while smaller than the bulk G-CN powder (2.72eV). It may indicate that the B-doped nanolayer partially contribute to the light absorption of the s-BCN film.

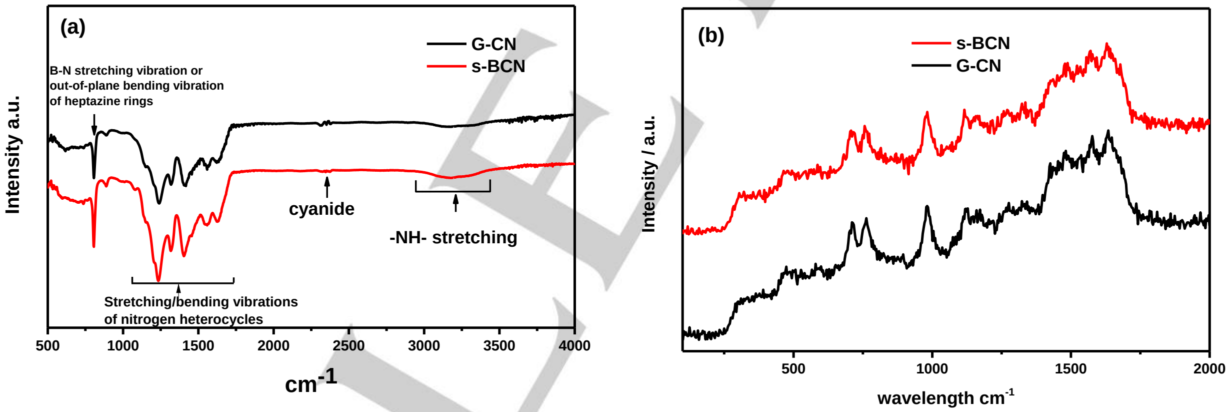

Figure S7 a) FTIR and b) Raman spectrum of G-CN and s-BCN

The FT-IR spectrum (Figure S7a) of s-BCN reveals a typical local structure of G-CN. The intense band at 806 cm^{-1} was attributed to the out-of-plane bending vibration characteristic of heptazine rings overlaped with B-N stretching vibration[6]. The bands at 1311 and 1226 cm^{-1} can be assigned to stretching vibration of C–N(–C)–C or C–NH–C units. The intense bands at 1633, 1558, 1456 and 1406 cm^{-1} represent typical stretching vibration modes of heptazine-derived repeating units. Raman spectroscopy allows the fine structure of G-CN and s-BCN to be examined in detail (Figure S7b). A series of peaks between 1200-1700 cm^{-1}, specifically (N(N=C=N), 1400 cm^{-1}; N(–CN), 1600 cm^{-1}; C(sp2), 1380 cm^{-1} and C(sp3), 1600 cm^{-1}) were attributed to C-N stretching vibrations [7]. The peak at 980 cm^{-1} can be attributed to the symmetric N-breathing mode of heptazine, whilst the peak at 690 cm^{-1} was assigned to the in-plane bending vibrations of the tri/heptazine C-N-C linkages[8]. All peaks therefore confirm that s-BCN has a typical local structure of G-CN.

WILEY-VCH

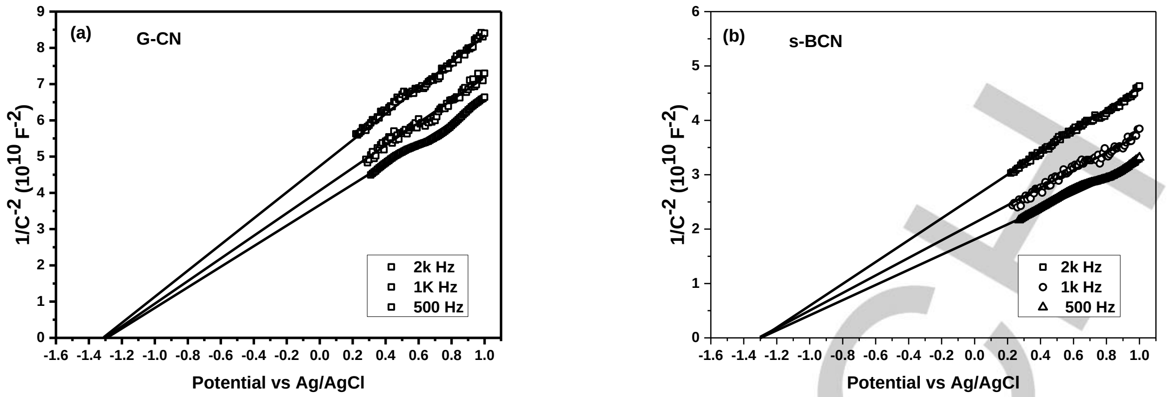

Figure S8 Mott-Schottky plots of a) G-CN and b) s-BCN at 2k, 1k and 0.5 k Hz frequencies.

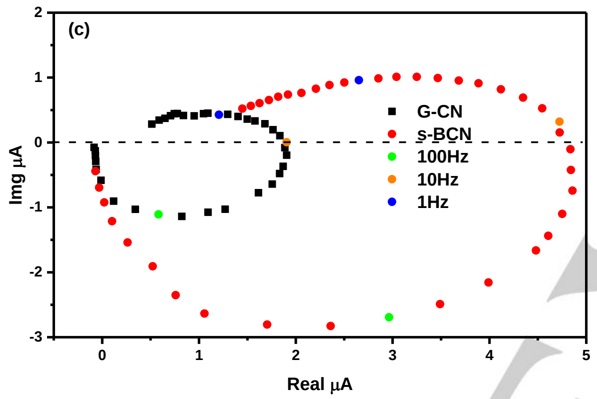

Figure S9 typical IMPS response of G-CN and s-BCN films at $0.1V_{Ag/AgCl}$

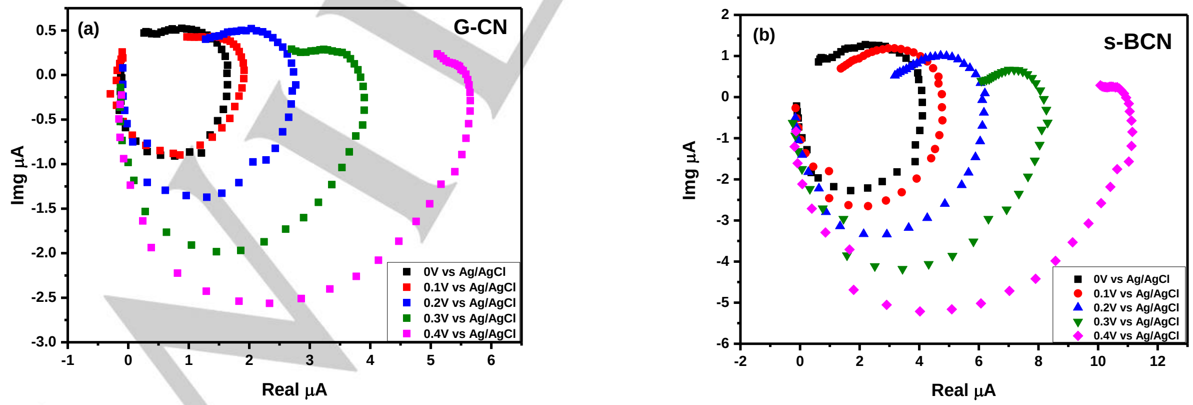

Figure S10 IMPS plots of a) G-CN and b) s-BCN

WILEY-VCH

A summary of PEC performance of CN based polymer photoanode in the absence of any sacrificial reagents has been shown in Table S1.

Table S1. Comparison of high performance CN based polymer photoanode toward PEC water splitting

CN photoanode	photocurrent density ($\mu A/cm^2$)	Light source	potential	IPCE	ref
g-CN	30.2	500W, >420nm	1.23V vs RHE	N/A	[9]
g-CN	20	AM 1.5	0.8V vs Ag/AgCl (0.1M KCl electrolyte)	N/A	[10]
g-CN	30	AM 1.5	1.55V vs RHE	<2.5% at 400nm	[11]
g-CN	63	AM 1.5	1.23 vs RHE	<2% at 400nm	[12]
Our work	103.2	AM 1.5	1.23 vs RHE	10% at 400nm	

Reference

[1] W. Luo, Z. Yang, Z. Li, J. Zhang, J. Liu, Z. Zhao, Z. Wang, S. Yan, T. Yu, Z. Zou, *Energy & Environmental Science* **2011**, *4*, 4046-4051.
[2] Q. Gu, Y. Liao, L. Yin, J. Long, X. Wang, C. Xue, *Applied Catalysis B: Environmental* **2015**, *165*, 503-510.
[3] D. J. Martin, K. Qiu, S. A. Shevlin, A. D. Handoko, X. Chen, Z. Guo, J. Tang, *Angewandte Chemie International Edition* **2014**, *53*, 9240-9245.
[4] Y. Wang, H. Li, J. Yao, X. Wang, M. Antonietti, *Chemical Science* **2011**, *2*, 446-450.
[5] M. Kawaguchi, T. Kawashima, T. Nakajima, *Chemistry of materials* **1996**, *8*, 1197-1201.
[6] S. K. Kisku, S. K. Swain, *Journal of the American Ceramic Society* **2012**, *95*, 2753-2757.
[7] aS. Yang, Y. Gong, J. Zhang, L. Zhan, L. Ma, Z. Fang, R. Vajtai, X. Wang, P. M. Ajayan, *Advanced materials* **2013**, *25*, 2452-2456; bA. C. Ferrari, J. Robertson, *Physical review B* **2000**, *61*, 14095.
[8] P. Larkin, M. Makowski, N. Colthup, *Spectrochimica Acta Part A: Molecular and Biomolecular Spectroscopy* **1999**, *55*, 1011-1020.
[9] J. Liu, H. Wang, Z. P. Chen, H. Moehwald, S. Fiechter, R. van de Krol, L. Wen, L. Jiang, M. Antonietti, *Advanced Materials* **2015**, *27*, 712-718.
[10] X. Wei, H. Jiang, Z. Liu, *RSC Advances* **2016**, *6*, 81372-81377.
[11] J. Bian, Q. Li, C. Huang, J. Li, Y. Guo, M. Zaw, R.-Q. Zhang, *Nano Energy* **2015**, *15*, 353-361.
[12] X. Lv, M. Cao, W. Shi, M. Wang, Y. Shen, *Carbon* **2017**, *117*, 343-350.

WILEY-VCH
